# Integrated mRNA–miRNA transcriptome profiling of blood immune responses potentially related to pulmonary fibrosis in forest musk deer

**DOI:** 10.3389/fimmu.2024.1404108

**Published:** 2024-05-30

**Authors:** Wen-Hua Qi, Li-Fan Hu, Yu-Jiawei Gu, Xiao-Yan Zhang, Xue-Mei Jiang, Wu-Jiao Li, Jun-Sheng Qi, Guo-Sheng Xiao, Hang Jie

**Affiliations:** ^1^College of Biological and Food Engineering, Chongqing Three Gorges University, Chongqing, China; ^2^College of Environmental and Chemical Engineering, Chongqing Three Gorges University, Chongqing, China; ^3^Chongqing Wanzhou NO. 3 Middle School, Chongqing, China; ^4^Department of Laboratory Medicine, Shenzhen Children’s Hospital, Shenzhen, China; ^5^Jinfo Mountain Forest Ecosystem Field Scientific Observation and Research Station of Chongqing, Chongqing Institute of Medicinal Plant Cultivation, Chongqing, China

**Keywords:** blood transcriptome, immune response, miRNA-mRNA network, signal pathway analysis, RT-qPCR, forest musk deer

## Abstract

**Background:**

Forest musk deer (FMD, *Moschus Berezovskii*) is a critically endangered species world-widely, the death of which can be caused by pulmonary disease in the farm. Pulmonary fibrosis (PF) was a huge threat to the health and survival of captive FMD. MicroRNAs (miRNAs) and messenger RNAs (mRNAs) have been involved in the regulation of immune genes and disease development. However, the regulatory profiles of mRNAs and miRNAs involved in immune regulation of FMD are unclear.

**Methods:**

In this study, mRNA-seq and miRNA-seq in blood were performed to constructed coexpression regulatory networks between PF and healthy groups of FMD. The hub immune- and apoptosis-related genes in the PF blood of FMD were explored through Gene Ontology (GO) and the Kyoto Encyclopedia of Genes and Genomes (KEGG) enrichment analysis. Further, protein–protein interaction (PPI) network of immune-associated and apoptosis-associated key signaling pathways were constructed based on mRNA-miRNA in the PF blood of the FMD. Immune hub DEGs and immune hub DEmiRNAs were selected for experimental verification using RT-qPCR.

**Results:**

A total of 2744 differentially expressed genes (DEGs) and 356 differentially expressed miRNAs (DEmiRNAs) were identified in the PF blood group compared to the healthy blood group. Among them, 42 DEmiRNAs were negatively correlated with 20 immune DEGs from a total of 57 correlations. The DEGs were significantly associated with pathways related to CD molecules, immune disease, immune system, cytokine receptors, T cell receptor signaling pathway, Th1 and Th2 cell differentiation, cytokine-cytokine receptor interaction, intestinal immune network for IgA production, and NOD-like receptor signaling pathway. There were 240 immune-related DEGs, in which 186 immune-related DEGs were up-regulated and 54 immune-related DEGs were down-regulated. In the protein-protein interaction (PPI) analysis of immune-related signaling pathway, *TYK2*, *TLR2*, *TLR4*, *IL18*, *CSF1*, *CXCL13*, *LCK*, *ITGB2*, *PIK3CB*, *HCK*, *CD40*, *CD86*, *CCL3*, *CCR7*, *IL2RA*, *TLR3*, and *IL4R* were identified as the hub immune genes. The mRNA-miRNA coregulation analysis showed that let-7d, miR-324-3p, miR-760, miR-185, miR-149, miR-149-5p, and miR-1842-5p are key miRNAs that target DEGs involved in immune disease, immune system and immunoregulation.

**Conclusion:**

The development and occurrence of PF were significantly influenced by the immune-related and apoptosis-related genes present in PF blood. mRNAs and miRNAs associated with the development and occurrence of PF in the FMD.

## 1 Introduction

Musk deer (*Moschus* spp.), a rare and critically endangered species endemic to China, Vietnam, and other Asian countries, has attracted the attention of the government and the international organizations concerned. More than 80% of the musk deer are distributed in China, the musk production of which accounted for 90% of the world musk output before the 1950s. Due to habitat destruction and killing musk deer for musk harvesting, the population of musk deer has declined rapidly since the 1950s. For this reason, all musk deer have been classified as a Grade 1 protected animal in China since 2002 (1). Musk was secreted by the odor glands of male musk deer, which has multiple medicinal effects such as neuroleptic, anti-inflammatory, anti-thrombotic, and anti-tumor in the medicine industry (2, 3). Moreover, musk has been widely used in the high-end perfume industry (4, 5). Forest musk deer (FMD; *Moschus Berezovskii*) is a species of musk deer, which is the most farmed musk deer species, followed by the alpine musk deer (AMD; *Moschus chrysogaster*) in China. At present, the musk mainly came from the captive FMD and AMD in China.

In order to make sustainable use of musk deer resources and reduce the killing of wild musk deer, artificial musk deer farms have been set up in China since 1958 (6). Although China’s artificial breeding of musk deer has been experienced for more than 60 years, the population of captive musk deer is increasing slowly, which is closely related to the high mortality caused by multiple diseases. Although the captive breeding of FMD has made great progress, its long-term breeding is hampered by multiple diseases, including pulmonary disease (7). In the animal farms, the common diseases of musk deer industry included abscess, pulmonary disease, gastroenteritis, and parasitic diseases, which were important constraints to the growth of China’s musk industry (8). Pulmonary disease, a respiratory disease is closely associated with dozens of diseases including pneumonia, tuberculosis, and pulmonary fibrosis (PF) (9–12). Pulmonary disease is a primary life-threatening for captive FMD. PF is a fatal disease of the respiratory system that affects the health and survival of animals accompanied by an overproliferation of fibroblasts and an inflammatory reaction (13–15). Bacterial pneumonia, especially PF, severely affected the survival of FMD in captivity (16). There are many causes for the formation of PF, among which infectious bacterial are the important causes (17). It has been reported that *Pseudomonas aeruginosa*, *Streptococcus pneumoniae*, *Staphylococcus aureus*, and *Streptococcus pneumoniae* can induce the progression of PF (17, 18).

Blood is an essential part of the immune system and also the first line of defense against infectious disease (19, 20). The changes of gene expression in blood cells were influenced by pathological changes of animal body (21). Therefore, immune system status can be monitored by blood transcriptome. Multiple pathogenic infections and immune system dysfunction have been reported to be associated with the development of pneumonia, phthisis, and PF (9, 22, 23). Analysis of the blood transcriptome also helps to identify immune genes and their signaling pathways. MiRNAs modulated RNA silencing and post-transcriptional gene expression regulation, which took part in the immune system, immune diseases, and apoptosis. Consequently, the purpose of the present study was to establish a dependable blood miRNA biomarker for the diagnosis of PF. As far as we know, there is no current systematic and comprehensive analysis of miRNA–mRNA regulatory networks based on PF and healthy blood groups derived from FMD. The building of potential miRNA–mRNA regulatory networks is going to help identify the full range of molecular mechanisms by which miRNA affects PF and may be used in the diagnosis of the disease.

The present study screened for unigenes and miRNAs that were differentially expressed, as well as immune-related genes and apoptosis-related genes in the PF and healthy peripheral blood of FMD by high-throughput sequencing. The hub immune- and apoptosis-related genes in the PF blood of FMD were explored through Gene Ontology (GO) and the Kyoto Encyclopedia of Genes and Genomes (KEGG) enrichment analysis. The mRNA–miRNA interaction network is important in regulating immune and apoptotic functions. The present study was aimed at constructing multiple protein–protein interaction (PPI) network of immune-associated and apoptosis-associated key signaling pathways in the PF blood of the FMD. DEmiRNAs may be involved in the regulation of hub immune- and apoptosis-related genes in the PF blood of FMD were identified by Spearman correlation analysis. Furthermore, the mRNA–miRNA regulatory network may help to understand the mechanisms involved in the development of PF blood in FMD.

## 2 Materials and methods

### 2.1 Sample collection

The sick FMDs were looked after by us all day long. We take blood as soon as they got worse. When the sick FMDs died, an autopsy was performed by veterinarian to determine the PF. The 17 FMD venous blood samples were taken at the Chongqing Institute of Medicinal Plant Cultivation in Chongqing, China (**Table 1**; **Supplementary Figure S1**). Fresh blood samples were promptly kept in RNAlater (Ambion Inc., Austin, TX, USA). The PF and healthy blood groups of FMD were taken between January 2021 and December 2022 in Chongqing, China. All animal experiments were approved by Chongqing Three Gorges University and Chongqing Institute of Medicinal Plant Cultivation. The collected blood samples are packaged, sealed, labeled, and immediately refrigerated at 4°C before being sent to the laboratory. For long-term storage, the blood should be rapidly frozen at −40°C immediately after collection and then stored at −80°C for no more than three months.

**Table 1:** Summary of sample information.

Sample	Age	Gender	Group
DAM2_2	2	Male	PF
DOM12_1	12	Male	PF
DOM12_2	12	Male	PF
DOM13	13	Male	PF
HAM1	2	Male	Health
HAM2	2	Male	Health
HAF2_2	2	Female	Health
HAM3	3	Male	Health
HAM4	4	Male	Health
DG10M	10	Male	PF
DG4FM	4	Female	PF
DG6FM	6	Female	PF
DG6M	6	Male	PF
HG13M	13	Male	Health
HG15FM	15	Female	Health
HG4.10FM	10	Female	Health
HG5M	5	Male	Health

### 2.2 mRNA and miRNA sequencing and differential expression analysis

The total RNA was extracted using TRIzol reagent and then processed with RNase-free DNase I. A whole RNA pool was collected the venous blood of 17 FMD individuals to create an mRNA library or a small RNA library by using an Illumina HiSeq2500 platform (24, 25). Principal components analysis (PCA) of mRNAs and miRNA expression were performed in the nine samples and eight samples, respectively. The known miRNAs were identified by aligning them against the miRBase (26), and the novel miRNAs were predicted by using miranda (27). Expression levels of genes and miRNAs estimated as FPKM (fragments per kilobase million) and TPM (transcripts per million) indices, respectively, which were used to determine DEGs and DEmiRNAs between PF and healthy blood groups. The unigenes with *P_adj_* < 0.05 and |log_2_(fold change) | >1.0 were taken as the threshold for DEGs. DEmiRNAs were identified with the thresholds of *p* < 0.05 and | log_2_ (fold change) | >2.0.

### 2.3 miRNA target gene prediction and enrichment analysis

The target genes of DEmiRNAs in the two groups were predicted by using RNA hybridization (28), MiRanda (29), and PITA (Probability of Interaction by Target Accessibility, 30), and the results from the three algorithms were intersected. The potential target genes predicted by the above two softwares were combined, and the intersecting components were included as a set of candidate target genes. DEGs and DEmiRNA target mRNAs were further used to fulfil the GO and KEGG enrichment analyses. The *p* < 0.05 was designated as the threshold for significance.

### 2.4 miRNA–mRNA network integration

A set of 1,811 immune-related genes was obtained from ImmPort. Additionally, 306 apoptosis-related genes were recognized from the molecular signature database (MSigDB), which were intersected with the DEGs of the PF and healthy blood groups to regard as immune DEG set and apoptotic DEG set. In the typical immune pathway of this study, these immune DEGs were uploaded to STRING and obtain their interaction information (31, 32). The key potential regulatory networks of immune-related DEGs (or apoptosis-related DEGs) in the immune pathway (or apoptotic pathway) were pictured using the cytoscape software. It has been reported that DEGs and DEmiRNAs have potential negative regulatory relationships. On the basis of this idea, we researched the expression correlation of immune DEGs (or apoptotic DEGs) and DEmiRNAs by using the PCC (Pearson correlation coefficient). The negatively coexpressed DEmiRNA–DEG pairs with PCC < −0.7 and *p* < 0.05 were screened to construct miRNA-gene networks.

### 2.5 Real-time fluorescent quantitative PCR

Six immune hub DEGs (*TLR2*, *TLR4*, *CXCL13*, *IL18*, *AKT1*, and *ITGB2*) and six immune hub DEmiRNAs (let-7f-5p, let-7d, miR-30b-3p, miR-25-5p, miR-149-5p, and miR-760) were chosen for differential expression analysis. The primers for six immune-related DEGs and six DEmiRNAs were provided in **Supplementary Table S1**. Each sample’s mRNA and miRNA was reverse transcribed using PrimeScript™ RT reagent Kit (TaKaRa) and a miScript II RT Kit (Qiagen, Hilden, Germany). Quantitative PCR of the immune DEGs and immune DEmiRNAs were performed by using TB GreenR Premix Ex TaqTM II (Takara) and miScript SYBR Green PCR Kit (Qiagen), respectively. GAPDH and U6 snRNA were used as reference gene and miRNA, respectively. The relative expression of six DEGs and DEmiRNAs was computed using the 2^−ΔΔCt^ method. The mean ± SE of three tests were presented in the data. Statistical significance was assessed by using *t*-tests as follows: *p ≤ 0.05 and **p ≤ 0.01.

## 3 Results

### 3.1 Overview of mRNA library of the two blood groups of healthy and BF

To investigate the changes of gene expression profiles in the PF pathological changes of FMD body and to compare their differences with healthy blood groups, nine cDNA libraries were built from the two blood groups of PF and healthy FMD. The unprocessed data were stored in the NCBI Sequence Read Archive (SRA) with the accession number PRJNA916839. After quality filtering, a total of 400,157,758 and 423,446,384 clean reads, representing a total of 62.12 and 80.09 Gb nucleotides, were generated for the PF and healthy blood groups, respectively (**Supplementary Table S2**). Approximately 86.33% of the clean reads was mapped to the FMD reference genome (33), with a match ratio ranging from 78.40 to 90.83% (**Supplementary Table S3**). After eliminating the low level of genes and transcripts, the reads were organized into 19,445 known genes and 4,907 annotated transcripts. A PCA was conducted, revealing that the samples from the PF and healthy blood groups formed two separate clusters based on the first principal component (PC1), which accounted for 66% of the variance (**Supplementary Figure S2**). This indicated that the sequencing data was suitable for further analysis. A mean of 11,884 (70.92%) and 12,229 (74.62%) unigenes with FPKM values greater than 0.5 were acquired from the PF and healthy blood group of FMD, respectively (**Supplementary Figure S3**). A detailed overview was described in **Table 2**.

**Table 2:** Transcripts and genes of the merged assembly.

Item	Transcripts	Genes
Number of sequences	78,239	24,352
Max length of sequence (bp)	154,753	101,907
Min length of sequence (bp)	132	132
Mean length (bp)	3,254.5	1,413.9
Total length (bp)	254,626,504 (254.63Mb)	34,432,182 (34.43Mb)
Contig N50 (bp)	9,363	2,025
GC content (%)	46	54
≥1000 bp	41,911	11,842

### 3.2 DEGs and functional annotation analysis

A total of 2,744 DEGs (1,657 upregulated and 1,087 downregulated genes) were identified in the PF blood groups compared to the healthy blood groups. In GO enrichment analysis, the DEGs in the PF blood group were found to be associated with various immune functions, including immune receptor activity (GO:0140375), adaptive immune response (GO:0002250), innate immune response (GO:0045087), regulation of immune response (GO:0050776), immune effector process (GO:0002252), immune system development (GO:0002520), regulation of immune system process (GO:0002682), activation of immune response (GO:0002253), cell activation involved in immune response (GO:0002263), immune response-regulating signaling pathway (GO:0002764), and myeloid cell activation involved in immune response (GO:0002275) (**Supplementary Table S4**). The 2744 DEGs were related to 116 KEGG pathways (**Supplementary Table S5**), in which the most common immune pathways were CD molecules, immune disease, the immune system, cytokine receptors, T-cell receptor signaling pathway, Th1 and Th2 cell differentiation, cytokine–cytokine receptor interaction, the intestinal immune network for IgA production, and NOD-like receptor signaling pathway.

### 3.3 Immune-related DEGs and apoptosis-related DEGs

**Figure 1:**
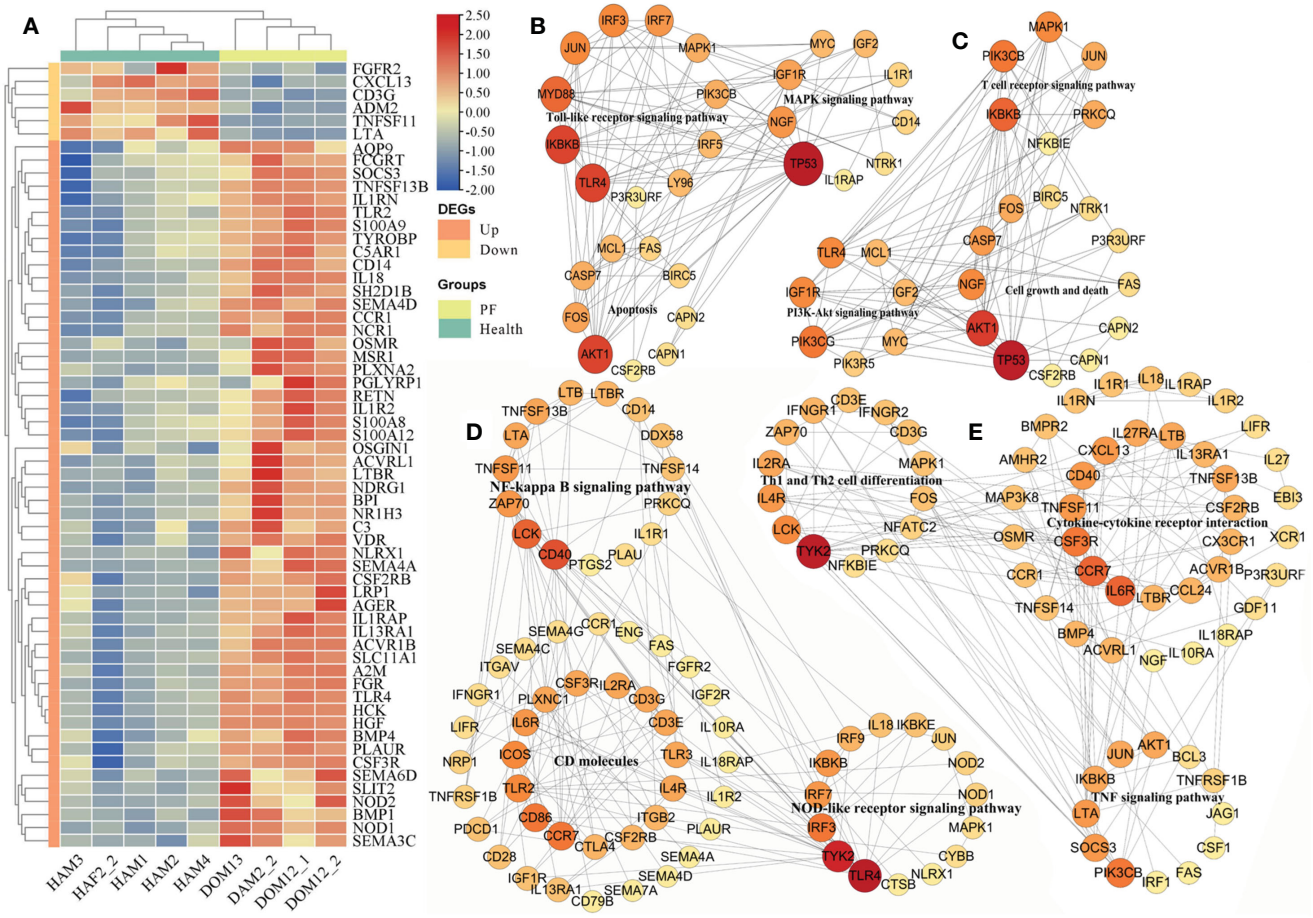
Analysis of immune-related DEGs in the PF and healthy blood of FMD. **(A)** Heatmap of immune-related DEGs. **(B, C)** The apoptosis-related DEGs interaction network in the key signaling pathway. **(D, E)** The immune-related DEGs interaction network in the key signaling pathway.

Among 2744 DEGs, 240 immune DEGs were identified and verified in the PF blood groups compared to the healthy blood groups, including 186 upregulated and 54 downregulated genes (**Supplementary Table S6**). The significant expression patterns of immune-related DEGs between PF and healthy blood groups are shown in **Supplementary Table S6**. In addition, we conducted a hierarchical cluster analysis of top 60 immune DEGs across the nine samples, which indicated that immune-related DEG expression levels in the PF groups could be robustly separated from those in the healthy groups (**Figure 1A**). The upregulated immune DEGs gathered into one group, and the downregulated immune DEGs gathered into another group. Similarly, PF and healthy blood groups were gathered separately (**Figure 1A**), thus highlighting gene expression differences between PF and healthy blood groups. The GO analysis revealed that the 240 immune DEGs were significantly enriched in BP including regulation of immune response, adaptive immune response, cell activation involved in immune response, leukocyte activation involved in immune response, cytokine production involved in immune response, immune system process, regulation of immune effector process, innate immune response, immune effector process; CC including receptor complex, extracellular region, and plasma membrane signaling receptor complex, and MF including immune receptor activity, cytokine receptor activity, signaling receptor binding, and molecular transducer activity (**Supplementary Table S7**). Collectively, these terms were all strongly linked to immunological function. Furthermore, the analysis of KEGG showed that these 240 immune DEGs were significantly enriched in various pathways related to the immune function. These pathways included the NOD-like receptor signaling pathway, Toll-like receptor signaling pathway, TNF signaling pathway, cytokine–cytokine receptor interaction, Th17 cell differentiation, Th1 and Th2 cell differentiation, CD molecules, immune system and immune disease (**Supplementary Table S8**).

In the PF blood group, 56 apoptosis-related DEGs (42 upregulated and 14 downregulated genes) were discovered. The significant expression patterns and distribution of the apoptotic DEGs between PF and healthy blood groups are shown in **Supplementary Table S9**. Furthermore, the analysis of GO revealed that these 56 DEGs related to apoptosis were significantly enriched in BP including regulation of mitochondrial membrane permeability involved in apoptotic process, positive regulation of apoptotic process, negative regulation of extrinsic apoptotic signaling pathway, apoptotic signaling pathway, apoptotic mitochondrial changes, leukocyte apoptotic process, glial cell apoptotic process, and execution phase of apoptosis; CC including proteasome accessory complex, organelle lumen, and membrane-enclosed lumen; and MF including cysteine-type endopeptidase activity involved in apoptotic signaling pathway, cysteine-type endopeptidase activity involved in the execution phase of apoptosis, cysteine-type endopeptidase inhibitor activity involved in apoptotic process, and peptidase activator activity involved in apoptotic process (**Supplementary Table S10**). An interesting point was that these terms were also related to the process of apoptosis. Furthermore, the KEGG analysis demonstrated that these 56 apoptosis-related DEGs were obviously enriched in apoptosis, cell growth, and death, B-cell receptor signaling network, NOD-like receptor signaling pathway, and NF-kappa B-signaling pathway (as shown in **Supplementary Table S11**).

### 3.4 Interaction network analysis of DEGs

In our analysis, we projected a total of 7,658 mRNA-mRNA pairs in the **BIPF** and healthy blood groups. The apoptosis-related DEGs in the apoptosis, Toll-like receptor signaling pathway, MAPK signaling pathway, T-cell receptor signaling pathway, cell growth and death, and PI3K-Akt signaling pathway were piped to STRING to create PPI networks (**Figures 1B, C**); the hub genes identified in one of the MCODE models in the PPI network including *TLR4*, *IKBKB*, *AKT1*, *MYD88*, *TP53*, *JUN*, *IRF3*, *IRF7*, *MAPK1*, *NGF*, and *IGF1R* major belonged to apoptosis, Toll-like receptor signaling pathway, and MAPK signaling pathway (**Figure 1B**); the other one MCODE model including *TP53*, *AKT1*, *IKBKB*, *PIK3CB*, *MAPK1*, *PIK3CG*, *IGF1R*, *TLR4*, *NGF*, and *CASP7*, which were mainly involved in T-cell receptor signaling pathway, cell growth and death, and PI3K-Akt signaling pathway (**Figure 1C**).

Among the KEGG pathways of immune-related DEGs in the PF and healthy blood groups, we observed that the majority of interaction networks were associated with CD molecules, cytokine–cytokine receptor interaction, NOD-like receptor signaling pathway, TNF signaling pathway, NF-kappa B-signaling pathway, immune system, immune disease, Th17-cell differentiation, Th1 and Th2 cell differentiation (**Figures 1D, E**; **Supplementary Figures S4**, **S5**). The immune-related DEGs in the NF-kappa B-signaling pathway, CD molecules, NOD-like receptor signaling pathway, cytokine–cytokine receptor interaction, Th1 and Th2 cell differentiation, and TNF signaling pathway were inputted into the STRING database to create PPI networks (**Figure 1D**); the hub genes identified in one of the MCODE models in the PPI network including *TLR4*, *TYK2*, *CD40*, *LCK*, *ZAP70*, *CD86*, *TLR2*, *CCR7*, *TLR3*, *IRF3*, *IRF7* major belonged to CD molecules, NF-kappa B-signaling pathway, and NOD-like receptor signaling pathway (**Figure 1B**); the other one MCODE model including *TYK2*, *IL6R*, *CCR7*, *CSF3R*, *CXCL13*, *LCK*, *IL4R*, *PIK3CB*, *SOCS3*, *IL2RA*, *LTA*, *CD40*, *TNFSF11*, and *IL27RA* were mainly in cytokine–cytokine receptor interaction, Th1 and Th2 cell differentiation, and TNF signaling pathway (**Figure 1E**). The most prevalent immune pathways associated with DEGs were involved in immune disease and immune system, in which interaction network of the related DEGs were built in **Supplementary Figure S4**; The hub genes, including *TLR4*, *LCK*, *ZAP70*, *CD86*, *ITGB2*, *ICOS*, *CD28*, *CD3E*, *CD40*, *TLR2* major belonged to the immune disease, functioned as core nodes in the regulatory network (**Supplementary Figure S4**); The hub genes, containning *PIK3CB*, *HCK*, *VAV1*, *ITK*, *TYK2*, *CCR7*, *FGR*, *IKBKB*, *IRF7*, *IRF3*, *PIK3CG*, *AKT1*, and *TLR3* were mainly in the regulatory network of immune system pathway (**Supplementary Figure S4**).

### 3.5 miRNA library construction and identification

**Figure 2:**
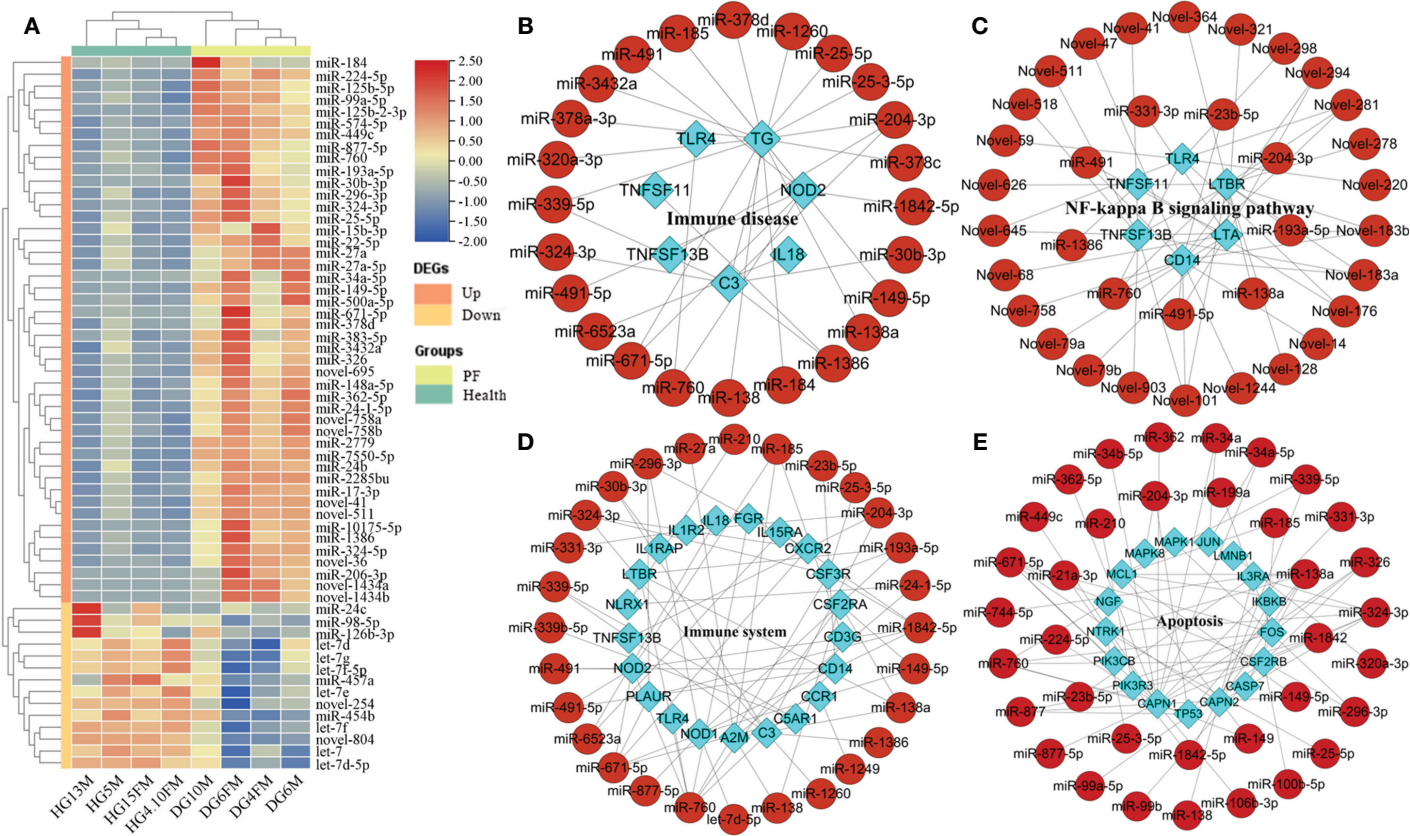
Analysis of DEmiRNAs in the PF and healthy blood of FMD. **(A)** Heatmap of DEmiRNAs. **(B–D)** DEmiRNA and target immune-related DEGs interaction network in the key signaling pathway. **(E)** DEmiRNA and apoptosis-related DEGs interaction network in the apoptosis pathway.

Eight short RNA libraries were created using blood samples from two groups: PF and healthy individuals of FMD. The counts of raw reads and clean reads of high-throughput sRNA were listed in **Supplementary Table S12**. Out of all the clean reads, about 99.08% were successfully aligned to the FMD reference genome. The unique match ratio ranged from 72.45% to 77.89% (**Supplementary Table S13**). Initially, the sRNA data were adjusted to mitigate the impact of technical noise. A PCA was conducted, revealing that the samples from the PF and healthy blood groups formed two independent clusters. This distinct cluster was mainly based on the PC1, which accounted for 47% of the total variance (**Supplementary Figure S6**). The unprocessed data have been stored in the NCBI SRA with the accession number PRJNA667837. The clean reads were annotated using the FMD reference genome and categorized into miRNAs, tRNAs, rRNAs, and other categories by performing a blast analysis using Rfam and miRBase. The unannotated RNAs were used to recognize novel miRNAs. The majority of miRNAs had lengths ranging from 21 to 23 nt, and the majority of the sequencing reads had a length of 22 nucleotides in the eight miRNA libraries. The research discovered a total of 3,206 miRNAs, out of which 1,407 miRNAs (700 known and 707 novel) were shown to be co-expressed in both PF and healthy blood groups. An average of 707 (69.77%) and 546 (64.12%) miRNAs with TPM > 0.5 were obtained in the PF and healthy blood group of FMD, respectively (**Supplementary Figure S7**). A heatmap of DEmiRNAs in the eight samples was plotted, which showed an obvious separation of DEmiRNAs and group clusters (**Figure 2A**).

### 3.6 DEmiRNAs and their target genes enrichment analysis

Three hundred fifty-six DEmiRNAs (332 upregulated and 24 downregulated miRNAs) were identified from a comparison of the PF and healthy blood sRNA libraries in the *M. berezovskii*. In the DEmiRNAs, 123 known and 233 novel mature miRNAs were identified. Among 356 DEmiRNAs, 53 immune-associated known DEmiRNAs and 55 immune-associated novel DEmiRNAs were identified and verified (**Supplementary Table S14**). The GO analysis of the DEmiRNA target immune genes were significantly enriched in the immune term including adaptive immune response, negative/positive regulation of immune response, activation of the immune response, regulation of immune system process, cytokine receptor activity, ligand-activated transcription factor activity, G protein–coupled peptide receptor activity, and activation of the immune effector process (**Supplementary Table S15**). KEGG analysis of these DEmiRNA-target immune genes that they were involved in the Toll-like receptor signaling pathway, antigen processing and presentation, RIG-I-like receptor signaling pathway, HIF-1 signaling pathway, Th1 and Th2 cell differentiation, TGF-beta signaling pathway, immune system, immune disease, and NF-kappa B-signaling pathway (**Supplementary Table S16**).

### 3.7 Immune-related DEGs and DEmiRNAs (apoptosis-related DEGs and DEmiRNAs) interaction analysis

The interaction networks of immune-related DEGs (or apoptosis-related DEGs) and DEmiRNAs, which regulated the FMD immune and apoptosis response, were predicted by the STRING and pictured by cytoscape software. The interaction network of DEmiRNAs with their target immune-related DEGs (or apoptosis-related DEGs) has also been constructed and demonstrated in the critical signal pathway (**Figure 2A**). In the immune disease, the network contained 97 molecules and 173 interactions, including 88 DEmiRNAs and nine immune DEGs (**Figure 2A**), in which the known DEmiRNAs, such as miR-149-5p, miR-185, miR-204-3p, miR-324-3p, miR-671-5p, and miR-760, related to immune disease, functioned as hub miRNAs in the regulatory network, and the target immune DEGs of these miRNAs included *TG*, *C3*, *NOD2*, *TLR4*, *TNFSF11*, *IL18*, and *TNFSF13B* as the core node genes. In the immune system, the network contained 132 molecules and 320 interactions, including 104 DEmiRNAs and 28 immune DEGs (**Figure 2B**), in which the known DEmiRNAs, such as let-7d-5p, miR-324-3p, miR-185, miR-204-3p, miR-193a-5p, miR-671-5p, miR-760, and miR-1842-5p related to immune system, functioned as hub miRNAs in the regulatory network, and the target immune DEGs of these miRNAs included *CXCR2*, *IL1R2*, *FGR*, *IL1RAP*, *IL15RA*, *C3*, *NOD1*, *NOD2*, *TLR2*, *TLR4*, *CCL3*, *IL18*, and *TNFSF13B* as the core node genes. In the Toll-like receptor signaling pathway, the network contained 31 molecules and 34 interactions, including 27 DEmiRNAs and four immune DEGs (**Supplementary Figure S8**), in which the known DEmiRNAs, such as miR-23b-5p, miR-491, miR-491-5p, and miR-760 related to Toll-like receptor signaling pathway, functioned as hub miRNAs in the regulatory network, and the target immune DEGs of these miRNAs included *TLR2*, *TLR4*, *CD14*, and *CCL3* as the core node genes.

In the NF-kappa B-signaling pathway, the network contained 42 molecules and 79 interactions, including 36 DEmiRNAs and 6 immune DEGs (**Figure 2B**), in which the known DEmiRNAs, such as miR-138a, miR-193a-5p, miR-204-3p, miR-23b-5p, miR-491, miR-491-5p, miR-331-3p, and miR-760 related to NF-kappa B-signaling pathway, functioned as hub miRNAs in the regulatory network, and the target immune DEGs of these miRNAs included *LTA*, *TLR4*, *CD14*, *TNFSF11*, *TNFSF13B*, and *LTBR*, as the core node genes. In the NOD-like receptor signaling pathway, the network contained 59 molecules and 72 interactions, including 54 DEmiRNAs and five immune DEGs (**Supplementary Figure S9**), in which the known DEmiRNAs, such as let-7d-5p, miR-149-5p, miR-185, miR-193a-5p, miR-204-3p, miR-23b-5p, miR-185, miR-210, miR-491, miR-491-5p, miR-671-5p, miR-877-5p, miR-760, and miR-1842-5p related to NOD-like receptor signaling pathway, functioned as hub miRNAs in the regulatory network, and the target immune DEGs of these miRNAs included *IL18*, *NOD1*, *NOD2*, *TLR4*, and *NLRX1*, as the core node genes. In the apoptosis signaling pathway, the network contained 136 molecules and 214 interactions, including 118 DEmiRNAs and 18 apoptosis-related DEGs (**Figure 2B**), in which the known DEmiRNAs, such as miR-138a, miR-877-5p, miR-149, miR-760, miR-138, miR-149-5p, miR-877, miR-671-5p,miR-339-5p, miR-326, miR-324-3p, miR-296-3p, miR-34a-5p, and miR-23b-5p, related to apoptosis signaling pathway, functioned as hub miRNAs in the regulatory network, and the target immune DEGs of these miRNAs included *MCL1*, *MAPK8*, *MAPK1*, *FOS*, *IL3RA*, *TP53*, *PIK3R3*, *PIK3CB*, *CASP7*, *CSF2RB*, *NTRK1*, *IKBKB*, and *JUN*, as the core node genes.

### 3.8 Validation of DEmRNAs and DEmiRNAs by qRT-PCR

**Figure 3:**
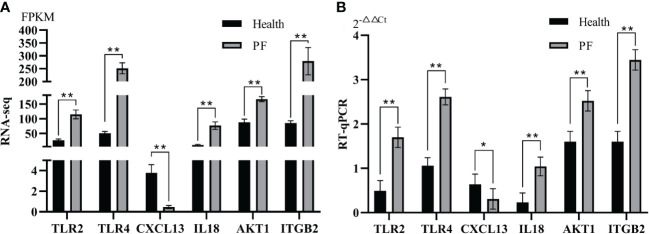
Verification of immune-related hub DEGs by RT-qPCR. **(A)** Immune-related hub DEGs expression in terms of FPKM as assessed by mRNA sequencing. **(B)** qRT-PCR analysis of six immune-related hub DEGs. Data represent the means ± SE. * represents a significant difference and ** represents a very significant difference.

**Figure 4:**
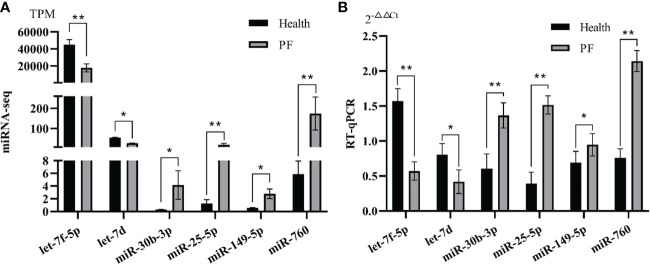
Verification of immune-related hub DEmiRNAs by RT-qPCR. **(A)** Immune-related hub DEmiRNAs expression in terms of TPM as assessed by RNA-seq. **(B)** qRT-PCR analysis of six immune-related hub miRNAs. Data represent the means ± SE. * represents a significant difference and ** represents a very significant difference.

In the study, six immune-related hub DEGs and six immune-related hub DEmiRNAs were chosen for qRT-PCR verification. The results showed that the FPKM of the six immune-related DEGs and the TPM of six DEmiRNAs from RNA-seq (*p* < 0.05; **Figures 3A**, **4A**) were consistent with those from the RT-qPCR data (**Figures 3B**, **4B**) based on the expression trends between the PF and healthy blood groups, which shown the high reliability of the RNA-seq results in the study.

## 4 Discussion

### 4.1 Innate immune response

Innate immunity is the first line of defense, which can mount resistance to reinfection. In the study, a differential expression analysis was conducted by utilizing mRNA-seq. 240 immune-related DEGs (54 downregulated and 186 upregulated) and 56 apoptosis-related DEGs (14 downregulated and 42 upregulated) were identified in the PF and healthy blood group of FMD. Genes rarely function alone, which form regulatory networks with other molecules to perform biological functions. At this point, immune-related DEGs and apoptosis-related DEGs were built to elucidate the regulatory relationships of DEGs. In the study, some immune-related DEGs (*CXCL13*, *CCL3*, *ISG20*, and *IL1R2*) were important to the innate immune response, which were upregulated in the PF blood groups. The interaction between *CXCL13* and its receptor (*CXCR5*) has been implicated in the pathogenesis of numerous diseases and the immune responses of healthy organisms (34, 35). The downregulation of *CXCL13* may recruit immune cells to the site of infection (34) and eliminate the proinflammatory response (36). *CLL3* gene highly expressed in several autoimmune diseases (37–39), which attracted a variety of leukocytes *in vitro* (40, 41) and thus played protective roles in all kinds of infectious diseases (42–45). Furthermore, it has been documented that *CCL3* can serve as a protective barrier against Chlamydia infection; conversely, *CCL3* deficiency would render an individual more vulnerable to contracting infectious diseases (38, 39). *ISG20*, a protein induced by interferon, participated in the innate immune response and has been found to play a role in inflammatory responses and viral infections (46). In addition, the upregulation of *ISG20*, an exonuclease gene, could inhibit the DNA and RNA virus replication (47). *NFKBIE*, *ISG20*, and *IL1R2* were crucial in regulating inflammation and host defense (47, 48). *CCL23*, *IRF1*, and *IRF7* were identified for immune-related DEGs, which had high expression level. The chemokine *CCL23* was secreted by different immune cells, which bound its receptor *CCR1* and involved in immune response (49, 50). *IRF7* could involve in regulation of type I interferon to against pathogens infections and the innate immune response (51). In our study, the upregulation of *ISG20*, *CCL23*, *IRF7* suggested that the strong innate immune response in the FMD had been activated by the PF.

To further clarify the function of immune DEGs and apoptotic DEGs, KEGG pathway enrichment analysis was performed. In our study, the NOD-like receptor signaling pathway, Toll-like receptor signaling pathway, cytokine–cytokine receptor interactions, CD molecules, immune disease, and immune system were significantly immune-related pathways, which was conducted to elucidate further the function of immune-related DEGs. An examination of the topology of interaction networks involving immune-related DEGs (apoptosis-related DEGs) revealed that the majority of key DEGs were members of the chemokine, interleukin, and *TLR* families. In this study, five *TLR* genes (*TLR1*, *TLR2*, *TLR3*, *TLR4*, and *TLR7*) were identified for immune DEGs, which participated in pathogens reorganization. In *TLRs*, *TLR1*, *TLR2*, *TLR4*, and *TLR7* were significantly upregulated in the PF, whereas *TLR3* was significantly downregulated in the PF. While *TLR5*, *TLR6* had no differences in the PF and healthy blood comparisons. *TLRs*, which stand for pattern recognition receptors, were identified as their role in recognizing pathogens and activating immune responses (52, 53). *TLR4*, *TLR5*, *TLR6*, *TLR7*, and *TLR8* can identify viral proteins and extracellular bacterial and fungal cell wall components (54), which were vital to the immune responses of the host against a variety of invading pathogens (53). *TLR2* and *TLR3* were able to induce the immune response by recognizing glycoproteins from various viruses (55, 56). *TLR4* participated the innate immunity of rodents against the respiratory syncytial virus (57). *TLR7* and *TLR8* have the capability to identify single-stranded RNA of viruses that are abundant in guanosine and uridine (58). The upregulation of *TLR2* and *TLR4*, which function as recognition receptors for the immune disease, played a crucial role in the innate immune system of FMD. Interleukin-18 (IL-18) was a highly potent pro-inflammatory cytokine that regulated innate and acquired immune responses and involved in the host’s defense against infections (59). In our study, *TLR2*, *TLR4*, and *IL18* were identified as hub gene, *TLR2* and *TLR4* were significantly upregulated, which promoted *IL18* production. This research was consistent with previous reports (60). Chemokines were very important for the role of the innate immune system and control the migration and positioning of immune cells, which were a defense against infection and inflammation. *CXCL13* and *CX3CR1* are both members of the chemokine family. *CXCL8*/*IL-8* was Proven to directly bind to Mycobacterium tuberculosis and potentially enhance the host resistance to infection (61). *IL-1R2* was an endogenous inhibitor that prevented the transduction of the *IL-1* signal (62). Prior research has demonstrated that *IL1R2* exhibited efficacy as an *in vivo* anti-inflammation and can cure various diseases (63, 64). It was the assertion that *IL1R2* secretion cannot eradicate the acute inflammatory response and restrict the inflammatory reaction to alleviate disease (65). Consequently, *IL1R2* upregulation may play a significant role in maintaining the physiological equilibrium of FMD inflammatory.

### 4.2 Adaptive immune response

The regulatory mechanisms of the adaptive immune system was regulated by T cells, B cells, and their antigen-specific receptors (*TCR* and *BCR*) (66). Innate immune response can resist the pathogen, while adaptive immune response can finally clear the infection and achieve long-lasting and highly specific protection and sustained by memory T cells (67, 68). *CD3E, CD3G, NFKBIE, TRBV4-1, TRBV12-3, TRAV26-1, IL4R*, and *IL2RA* were found to be enriched in the Th1 and Th2 cell division pathways, respectively, in the study. *TRAV14DV4*, *TRAV38-2DV8*, and *TRDC* were also significantly downregulated in the PF blood groups in the study. The TCR family, comprising *TRBV4-1, TRBV12-3, TRAV26-1, TRAV14DV4*, *TRAV38-2DV8*, and *TRDC*, was essential for adaptive immune initiation and facilitated the recognition of an array of antigens (69). *CD3E* and *CD3G* were present on the surface of T lymphocytes, which performed important functions in the adaptive immune response (70). *IL4R* was expressed on eosinophils, macrophages, lung fibroblasts, T and B lymphocytes, which was over-expressed and played significant role in the immune system (71–74). *IL2RA* is mainly expressed on mature T cells and some other activated hematopoietic cell, which played essential roles in immune regulation of multiple diseases (75).

A variety of interleukins (*IL2*, *IL4*, *IL5*, *IL6*, *IL7*, *IL10*, *IL13*, *IL14*, and *IL21*) were found to participate in the proliferation and differentiation of B cells (76–80). In the study, only *IL18*, *IL27*, and *IL36a* had significantly different expression and were significantly upregulated in the PF blood groups. *IL18* regulated both Th 1 and Th2 responses, which acted synergistically with *IL12* in the Th1 paradigm, whereas *IL18* with *IL2* and without *IL12* it can induce Th2 cytokine production from CD4^+^ T cells, natural killer (NK) cells, NKT cells, as well as from Th1 cells (81). *IL-27*, a member of the cytokine superfamily *IL-6*/*IL-12*, significantly regulated immune response (82). *IL-27* was discovered to play a broad anti-inflammatory function in infectious and chronic immune-mediated diseases (83–85). *IL36* is implicated in both the development and advancement of inflammatory and fibrotic disorders (1–7). Indeed, emerging evidence indicated that *IL-36* could mediate the relationship between inflammation and fibrosis (86–88). *IL-36a* was a members of the *IL36* cytokine family (89, 90). In addition to stimulating humoral immunity, *IL27* could suppressed T-cell responses in autoimmune conditions and thus induced inflammation and cell death in certain situations (91). *IL-18* is a crucial factor in facilitating the synthesis of specific cytokines (92, 93). The observation that the expression of *IL18* and the receptor of *IL18* (*IL18RAP*) was significantly increased in the PF blood group compared to the healthy blood group and the expressions of the two genes were negative. More research is required to determine whether the down expression of *IL18* affected the effectiveness of the immune responses.

### 4.3 Integrated analysis of DEGSs–DEmiRNA

MiRNAs regulated many cellular processes and signaling pathways, including embryogenesis, cell proliferation and differentiation, apoptosis, and disease onset. In doing so, miRNAs functioned as potent inhibitors of protein translation via the degradation of mRNAs (94, 95). Nevertheless, the hub miRNAs accountable for the aberrant regulation of immune response and apoptosis in the PF blood group of FMD still need to be further study. In our research, total 282 DEmiRNAs were targeted by the 217 immune DEGs with immunity, and 39 apoptotic DEGs were identified as miRNAs associated with both immunity and apoptosis. A total of 25 genes shared by immune-related DEGs and apoptosis-related DEGs that were identified in the PF and healthy blood groups. DEmiRNA–DEGs interaction networks were constructed based on the principle that miRNAs restrained protein translation by binding to the 3′UTR of target mRNAs and degrading them. In the study, the immune DEGs-DEmiRNA (and apoptotic DEGs-DEmiRNA) interaction network was first constructed in the PF blood group of FMD. In the apoptosis-related DEG–DEmiRNA network of apoptosis pathway, elevated expression of 42 apoptosis-related DEmiRNAs could induce the down expression of *MAPK1*, *MAPK8*, *TP53*, *PIK3R3*, and *NTRK1*, while the above 42 apoptosis-related DEmiRNAs could induce the upregulation of *IL3RA*, *CAPN1*, *CASP7*, *LMNB1*, *FOS*, *NGF*, *AKT1*, *JUN*, *PIK3CB*, *MCL1*, *CAPN2*, *CSF2RB*, and *IKBKB* (**Figure 2E**). In the immune-related DEG–DEmiRNA network of Toll-like receptor signaling pathway, elevated expression of miR-760, miR-23b-5p, miR-491, and miR-491-5p could induce the down expression of *CCL3*; while the above 4 apoptosis-related DEmiRNA could induce the upregulation of *CD14*, *TLR2*, and *IL18* (**Figure 2D**). In the immune-related DEG–DEmiRNA network of immune disease pathway, elevated expression of 24 immune-related DEmiRNAs could induce the down expression of *CCL3* and *TNFSF11*, while the above 24 immune-related DEmiRNAs could induce the upregulation of *TLR2*, *NOD2*, *TLR4*, *C3*, *TG*, *IL18*, *TNFSF13B*, and *TG* (**Figure 2B**). In the immune-related DEG–DEmiRNA network of immune disease pathway, elevated expression of miR-331-3p, miR-491, miR-491-5p, miR-760, miR-23b-5p, miR-1386, miR-204-3p, miR-193a-5p, and miR-138a could induce the down expression of *TNFSF11* and *LTA*; while the above nine immune-related DEmiRNAs could induce the upregulation of *CD14*, *TLR4*, *TNFSF13B*, and *LTBR* (**Figure 2B**). In the immune-related DEG–DEmiRNA network of immune system pathway, downregulated expression of let-7d-5p could induce the upregulated expression of *TNFSF11* and *LTA*, while the above nine immune-related DEmiRNAs could induce the upregulation of *CD14*, *TLR4*, *TNFSF13B*, and *LTBR* (**Figure 2C**). The integrated analysis of DEmiRNA–DEG interaction networks results indicated that most hub mRNAs are members of the chemokine, interleukin, and *TLR* families. It was confirmed that upregulation of hub mRNAs, including *CCL4*, *CXCL10*, *TLR2*, *TLR4*, and *TLR7*, occurred in the PF blood groups.

Investigations into the role of miRNAs in the pathogenesis of PF are still rarely. The differential expression of let-7f-5p between the PF and healthy blood groups was identified in the study, suggesting that let-7f-5p may serve as a biomarker for the investigation of the PF mechanism. This result was accordance with previous report (96). It has been reported that the let-7 family miRNAs were demonstrated to participate in the regulation of PF (97). These studies further support the hypothesis that PI3K and the let-7 family may have significant functions in the PF. It has been reported that Let-7f-5p prevents the PF by modulating cellular reactive oxygen species, mitochondrial DNA damage, and cell apoptosis (98). A prior investigation has validated the notion that the target genes of let-7f could enhance the transcriptional program of PF in a model of lung fibrosis induced by bleomycin (99). Notably, the target gene PIK3CA of let-7f-5p was an essential element of the PI3K/Akt pathway, which was instrumental in the pathogenesis of PF (14). It has been demonstrated that vascular endothelial growth factor, reactive oxygen species, and COX2 are involved in the PF in the downstream of the PI3K/AKT signaling pathway (100, 101). It has been believed that COX-2 was shown to regulate the expression of Fas receptor in pulmonary fibroblasts (102). Notably, we found that the downregulated let-7f-5p might induce the upregulation of SLC6A4, POR, and ATP13A3. The results of the integrated analysis of DEmiRNA–DEGs indicated that 29 miRNAs (18 upregulated and 11 downregulated) and 267 genes) formed miRNA-target gene pairs (**Figure 2**), these complex network of which potentially regulate neuronal cell proliferation, immune cell death, epilepsy, neurodevelopmental disorders, and Wnt/β-catenin and PTEN signaling.

## 5 Conclusions

In conclusion, the development and occurrence of PF were significantly influenced by the immune-related and apoptosis-related genes present in PF blood. mRNAs and miRNAs associated with the development and occurrence of PF in the FMD were investigated using RNA-seq technology in this study. It was possible to establish the interaction network between immune-related DEGs and DEmiRNAs (apoptosis-related DEGs and DEmiRNAs) by contrasting the profiles and functional analyses of DEmiRNAs and DEGs in the PF and healthy blood of FMD. We obtained 240 immune-related DEGs by RNA-seq, with 186 upregulated and 54 downregulated immune-related DEGs in the PF blood group compared to the healthy blood group. According to functional enrichment analysis, several immune-related pathways and terms were enriched with immune-related DEGs. Fifty-six apoptosis-related DEGs were obtained in the PF and healthy blood groups of FMD. As determined by functional enrichment analysis, apoptosis-related DEGs were enriched in several immune-related terms and pathways. Based on our findings, a gene set consisting of *TYK2*, *TLR2*, *TLR4*, *IL18*, *CSF1*, *CXCL13*, *LCK*, *ITGB2*, *PIK3CB*, *HCK*, *CD40*, *CD86*, *CCL3*, *CCR7*, *IL2RA*, *TLR3*, and *IL4R* could potentially function as immunoassay markers for the purpose of monitoring and evaluating the immune status of FMD. By examining networks of immune-related DEGs and DEmiRNAs (apoptosis-related DEGs and DEmiRNAs), our research will offer fresh perspectives on the molecular mechanisms that regulate the progression of PF.

## Data availability statement

The datasets presented in this study can be found in online repositories. The names of the repository/repositories and accession number(s) can be found in the article/**Supplementary Material**.

## Ethics statement

The animal study was approved by The Ethics Committee of Chongqing Three Gorges University and Chongqing Institute of Medicinal Plant Cultivation. The study was conducted in accordance with the local legislation and institutional requirements.

## Author contributions

W-HQ: Conceptualization, Investigation, Project administration, Supervision, Writing – original draft. L-FH: Data analysis, Investigation, Methodology, Software, Writing – original draft. Y-JG: Data analysis, Investigation, Visualization, Writing – review & editing. X-YZ: Investigation, Methodology, Software, Writing – review & editing. X-MJ: Data curation, Resources, Writing – review & editing. W-JL: Data curation, Formal analysis, Methodology, Software, Writing – review & editing. J-SQ: Conceptualization, Investigation, Project administration, Supervision, Writing – review & editing. G-SX: Conceptualization, Investigation, Project administration, Supervision, Writing – review & editing. HJ: Conceptualization, Funding acquisition, Supervision, Writing – review & editing, Project administration.
