# Supplementary figures and images for: Integrated mRNA–miRNA transcriptome profiling of blood immune responses potentially related to pulmonary fibrosis in forest musk deer
Source: Front Immunol. 2024 May 30;15:1404108. doi: 10.3389/fimmu.2024.1404108 (PMC11169664; doi:10.3389/fimmu.2024.1404108)

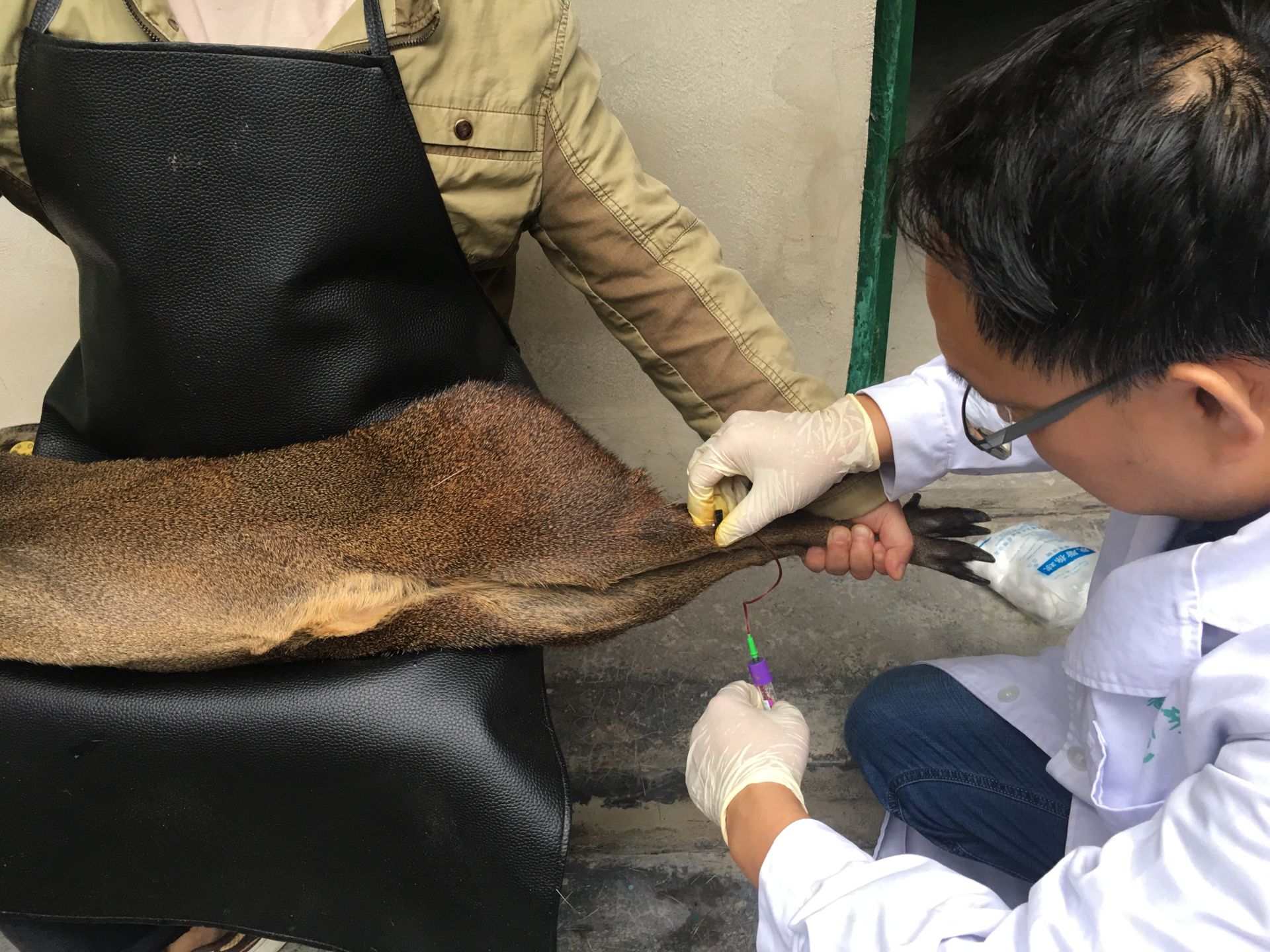

Supplement: Supplementary file 2 [file Image_1.jpeg]

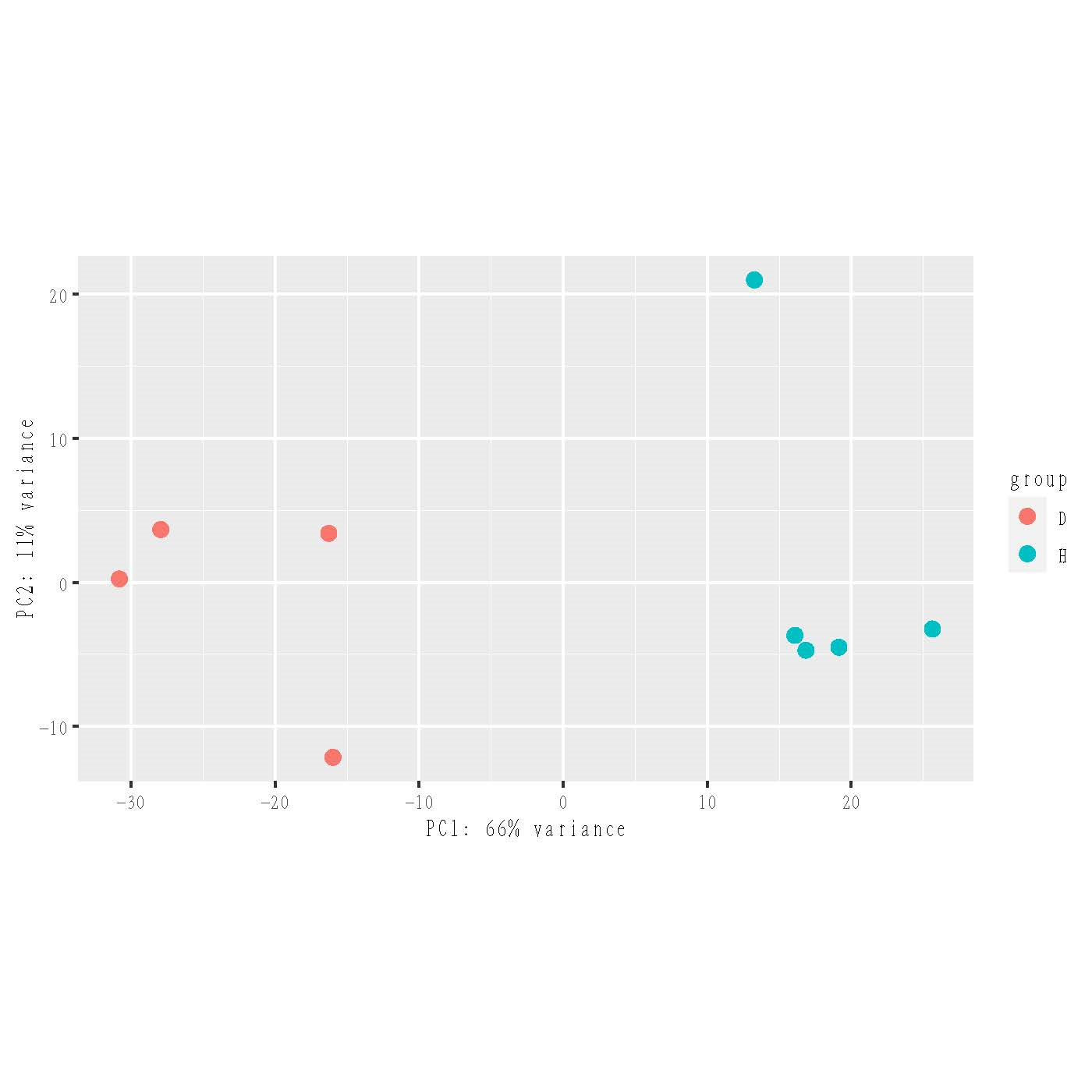

Supplement: Supplementary file 3 [file Image_2.png]

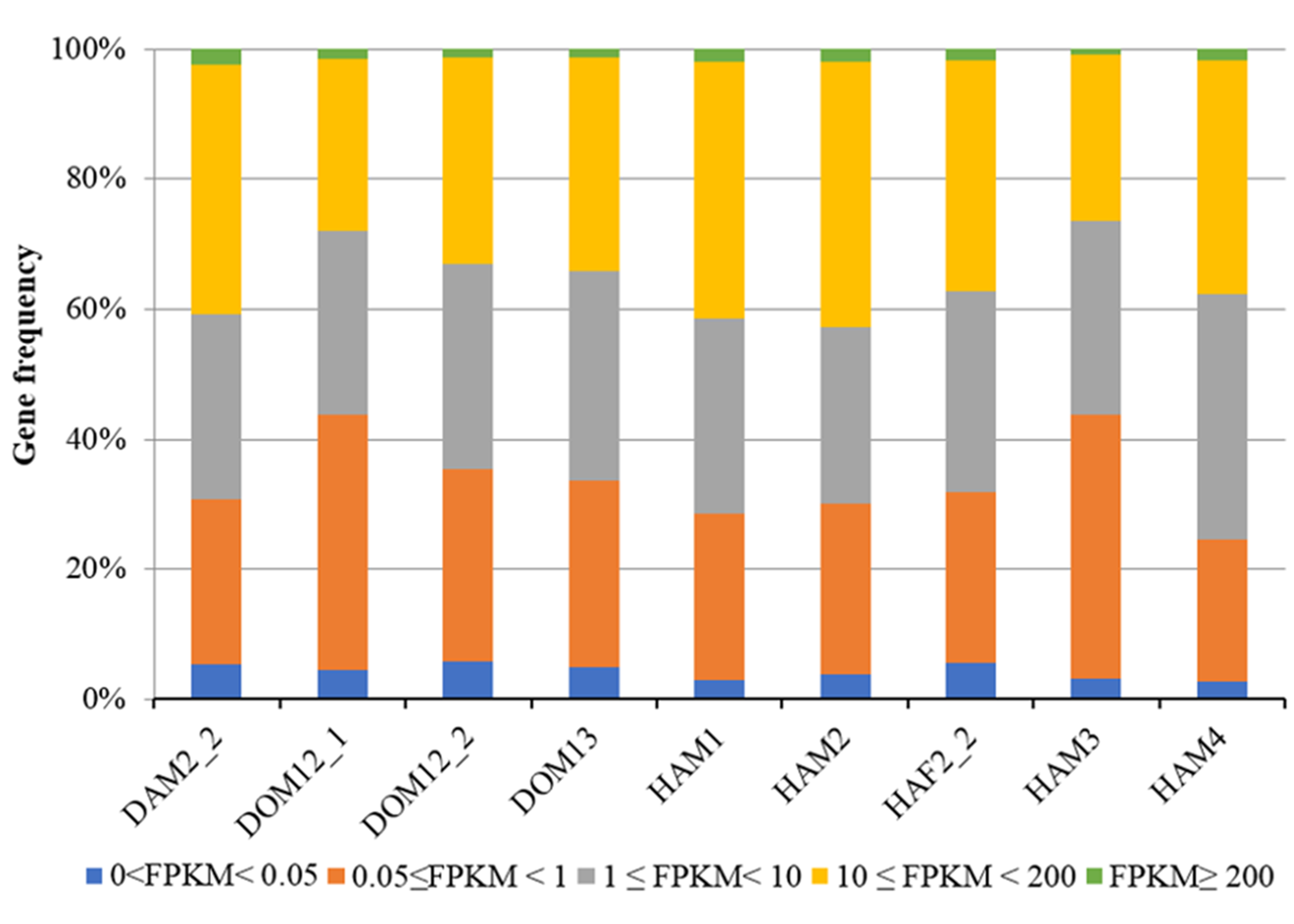

Supplement: Supplementary file 4 [file Image_3.png]

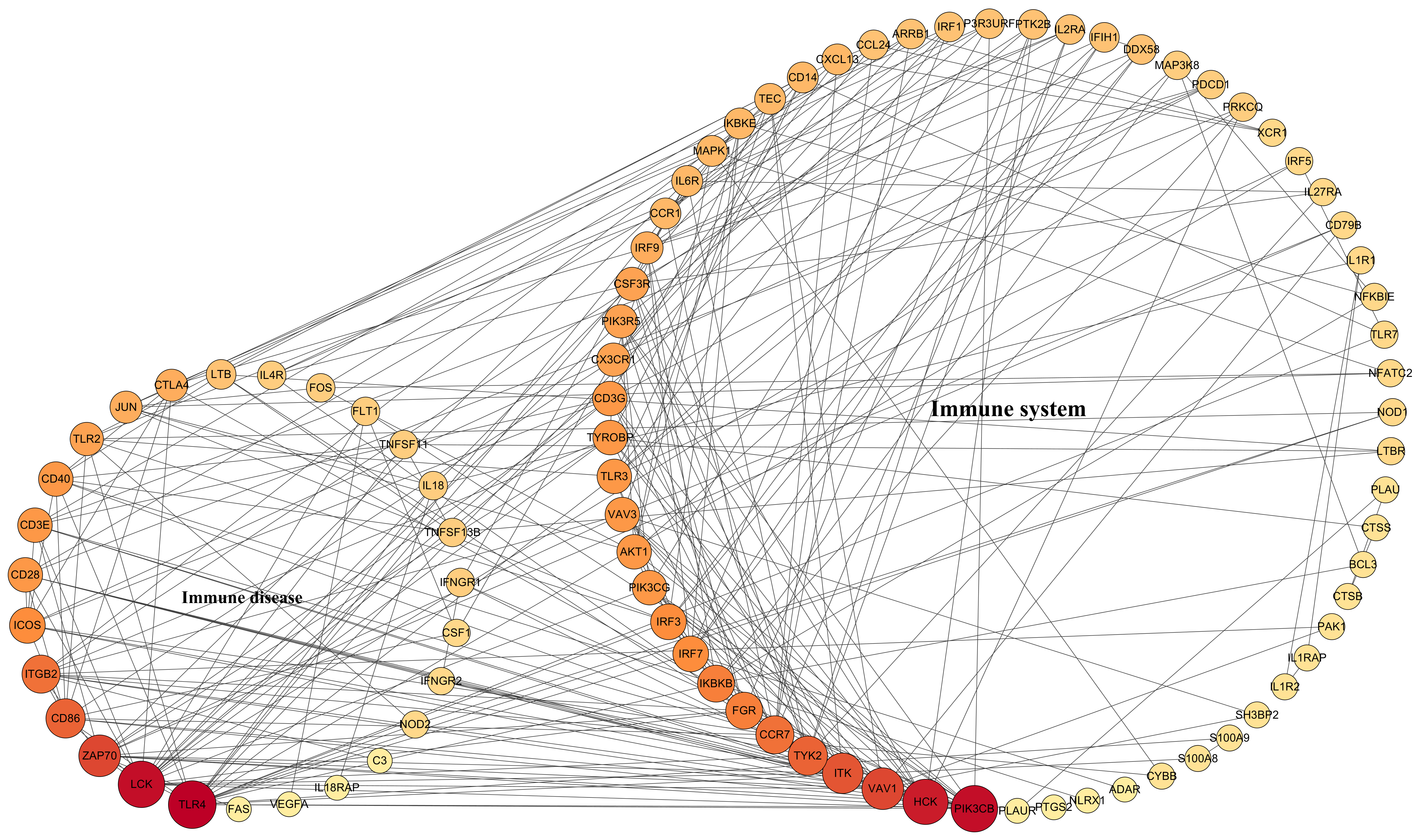

Supplement: Supplementary file 5 [file Image_4.png]

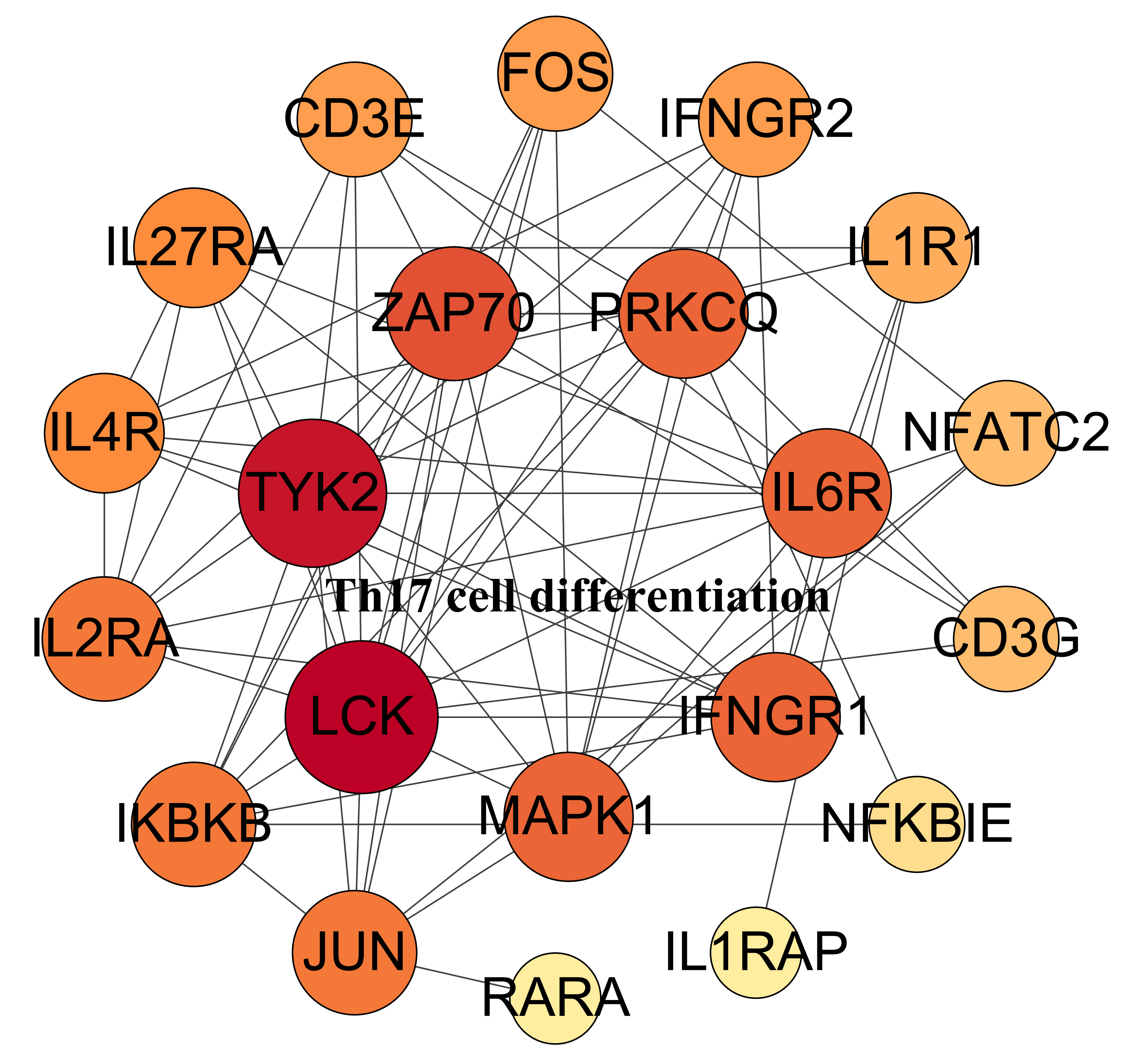

Supplement: Supplementary file 6 [file Image_5.png]

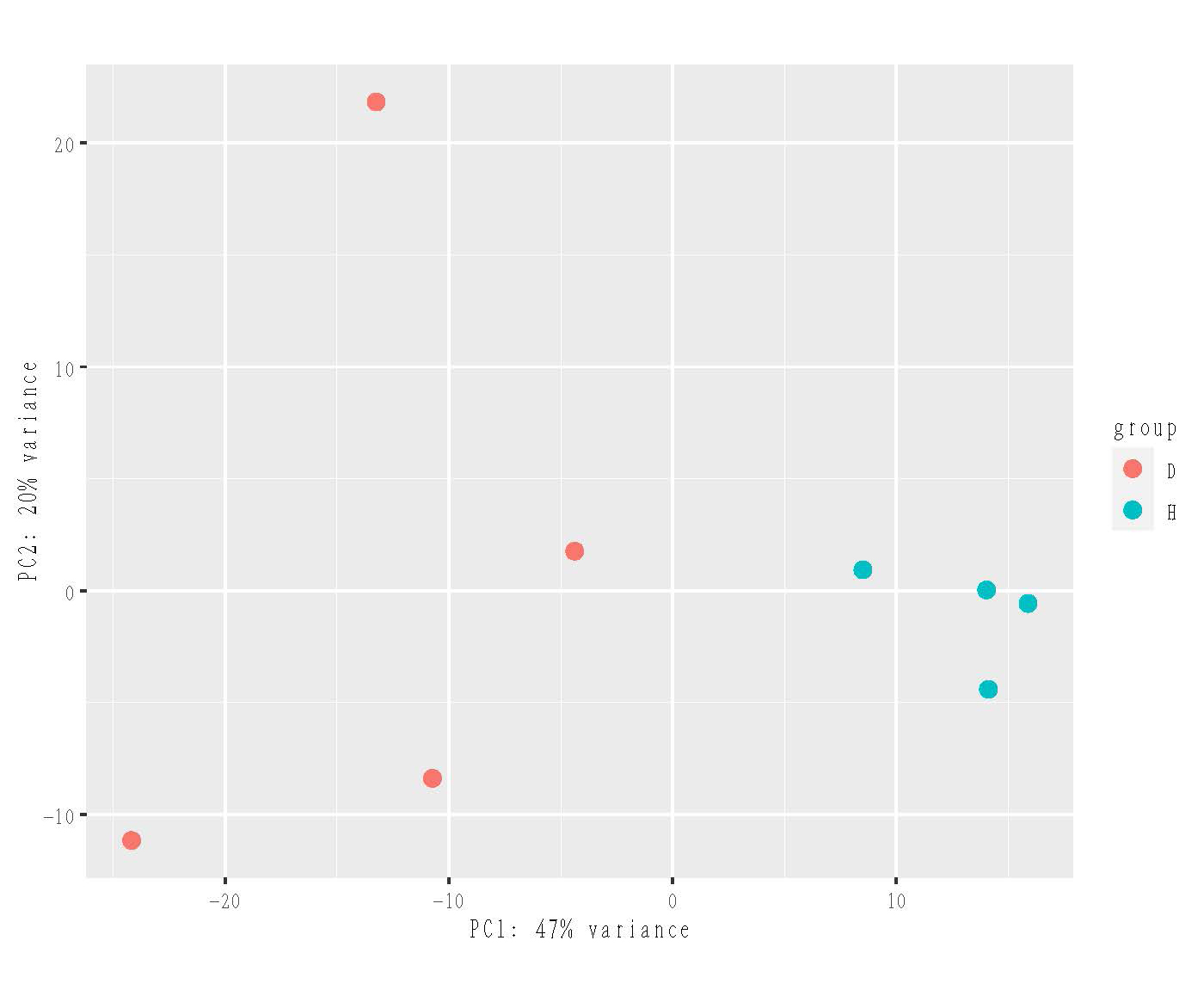

Supplement: Supplementary file 7 [file Image_6.jpeg]

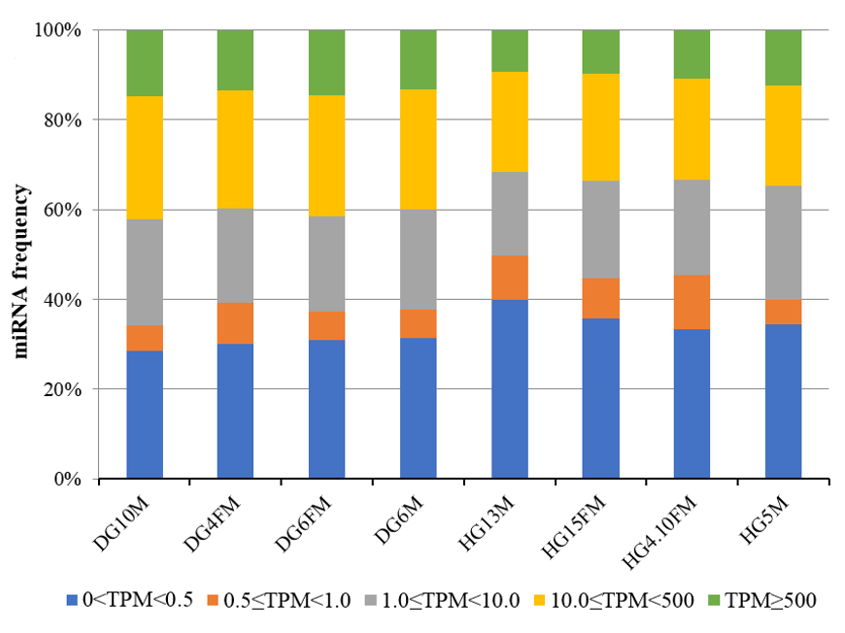

Supplement: Supplementary file 8 [file Image_7.png]

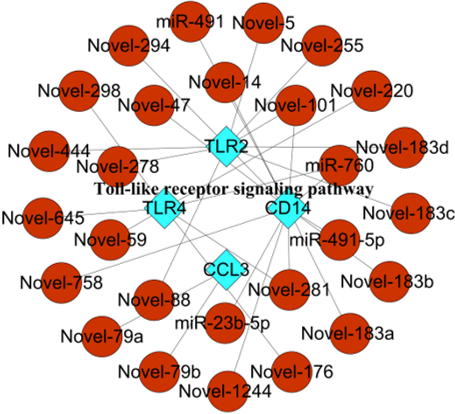

Supplement: Supplementary file 9 [file Image_8.png]

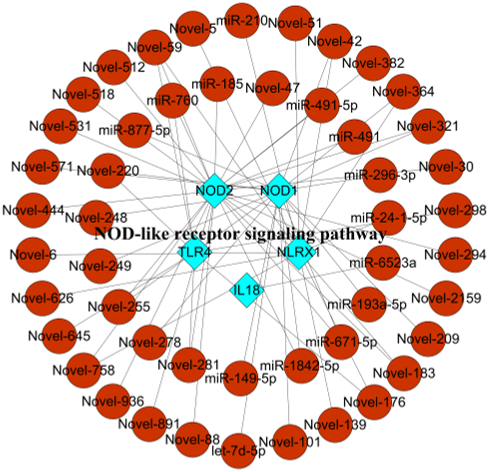

Supplement: Supplementary file 10 [file Image_9.png]
